# SuperCT: a supervised-learning framework for enhanced characterization of single-cell transcriptomic profiles

**DOI:** 10.1093/nar/gkz116

**Published:** 2019-02-25

**Authors:** Peng Xie, Mingxuan Gao, Chunming Wang, Jianfei Zhang, Pawan Noel, Chaoyong Yang, Daniel Von Hoff, Haiyong Han, Michael Q Zhang, Wei Lin

**Affiliations:** 1 University of Texas at Dallas. Department of Molecular and Cell Biology, Richardson, TX, USA; 2 Xiamen University, Department of Chemical Biology, Xiamen, Fujian, China; 3 National Engineering Research Center for Miniaturized Detection System, Northwest University, Xi’an, Shaanxi, China; 4 Translational Genomics Research Institute, Molecular Medicine Division, Phoenix, AZ, USA; 5 MOE Key Laboratory of Bioinformatics; Bioinformatics Division, Center for Synthetic & Systems Biology, BNRist and School of Medicine; Tsinghua University, Beijing, China; 6 Hunan Provincial Key Lab of Emergency and Critical Care, Hunan People’s Hospital, Changsha, Hunan, China

## Abstract

Characterization of individual cell types is fundamental to the study of multicellular samples. Single-cell RNAseq techniques, which allow high-throughput expression profiling of individual cells, have significantly advanced our ability of this task. Currently, most of the scRNA-seq data analyses are commenced with unsupervised clustering. Clusters are often assigned to different cell types based on the enriched canonical markers. However, this process is inefficient and arbitrary. In this study, we present a technical framework of training the expandable supervised-classifier in order to reveal the single-cell identities as soon as the single-cell expression profile is input. Using multiple scRNA-seq datasets we demonstrate the superior accuracy, robustness, compatibility and expandability of this new solution compared to the traditional methods. We use two examples of the model upgrade to demonstrate how the projected evolution of the cell-type classifier is realized.

## INTRODUCTION

Recent advances in single-cell RNA-seq (scRNA-seq) techniques make it possible to profile the RNA transcript abundance in a single cell, which enables us to reveal its identity. The mainstream scRNA-seq analytical methods utilize dimensional reduction (DR) and unsupervised clustering (UC) algorithms to initiate the analyses. UC provides the mathematical aggregation based on some cell grouping measures and DR facilitates the data visualization (DV) of the clustering result by ‘projection’. The putative subpopulations of cell types are thus identified with the enriched canonical signature signals. Nonetheless, this canonical workflow has its limitations. First, the cell types were not actually characterized by single-cell but by cluster. For each cell type in the sample, it always requires a certain number in order to form a discernable cluster. Second, the layout of cells by DR and the resultant clustering are model- and parameter-dependent. For example, the different distance/similarity metrics could result in different clustering effects (1–7). Kiselev *et al.* also have addressed the challenge of choosing optimal parameters and try to use a consensus matrix of multiple clustering results to optimize the final solution (8). Moreover, the cell-type assignment of these clusters highly relies on the investigator's familiarity with its signature molecules. Without sufficient background knowledge, the cell type, especially the rare types in a sample will be hard to identify, even though it could play critical roles. In this study, we aim to develop a new workflow that bypasses the clustering step and directly assign the cell type to each individual cell with less hassle on model selection or cluster interpretation.

Supervised classifier (SC) has been widely used in the automatic image classification (9–11). Ramo *et al.* developed CellClassifier based on the pixel intensities of cell imaging (12). However, using only morphological information is inadequate to find a definite answer because the identity of a single cell is mostly defined by its functional molecules rather than by how it looks. The genome-wide mRNA profiling provides more than enough information to discern its identity. SC3 method includes Support Vector Machine (SVM), a supervised learning component, which makes it a hybrid solution of UC and SC (8). Even though, the use of SVM is based on the cluster ID of the current dataset rather than the global learning of the features of the meaningful cell types. The cell-type classification somewhat resembles the image recognition in terms of high-dimensional data transformation and classification. Using a globally trained SC model, the user may easily solve the cell-type classification problem in one single step. Some typical challenges in scRNA-seq analyses, such as, the signal dropouts are like the dead pixels of images, won’t necessarily impede from recognizing them. All these facts make SC model not only a potential solution for cell-type classification but also an efficient and robust one.

In order to train the model and characterize cell types in a more efficient fashion at the user end, we hereby propose a non-linear SC model to predict cell types. The outperformance of the non-linear algorithm such as tSNE in the scRNA-seq feature space has suggested the non-linearity of the cell-type classification problem and the potential of non-linear classifier models such as SVM and Artificial Neural-Network (ANN). Unlike SC3 using the cells of the current study for local supervised training using SVM, we incorporate the total Mouse-Cell-Atlas (MCA) datasets (13) and other large-scale annotated single-cell datasets for the global training of the ANN model. Moreover, using the strategy of online learning, the ANN model can continuously optimize the performance and adapt itself to the prediction tasks in a specific sample context using the training dataset generated from the similar background. By increasing the output nodes and applying the online learning and the transfer learning, we are able to efficiently expand the cell-type catalog for a broader scope of characterization task. These are the extra benefits of the ANN as one of the SC options. In this paper, we extensively examine the utility, the reliability, the compatibility and the expandability of the SuperCT framework and demonstrate with a few explicit examples on how to characterize cell types and gain unprecedented insights of cell biology.

## MATERIALS AND METHODS

### Implementation of the SuperCT artificial neural network

The artificial neural-network structures and the learning algorithms are implemented using Keras API. We design a fully connected ANN model for the cell-type classification. The inputs are the binary signals of 16 013 genes that are homologous between human and mouse. We include these homologous genes to adapt the model to the application of both human and mouse study in this paper. To enhance the compatibility across different scRNA-seq platforms, we convert the digital expression values to the binary values, which means the genes are either present or absent in the cells. As seen in most of the flow-cytometry analyses, the present/absent of the signature gene provides adequate information to discern most of the known cell types. Therefore, it is believed that the dominant cell-type information is preserved after the binary transformation. Also, the binary signal input is compatible across most of the Unique-Molecular-Index-based (UMI) scRNA-seq platforms with the robust performance of the cell-type classification.

The input layer is connected to a hidden dense layer with 200 neurons and the first layer is fully connected to the next 100 neurons, respectively, using ReLU (Rectified Linear Unit) activation functions. Two random neuron dropouts (dropout rate at 0.4) occur after each layer in order to control over-fitting. The number of the output nodes corresponds to the number of the cell types in the catalog, which is 30+1 for v1m/v1h and 37+1 for v2m respectively. As the sample sizes of the different cell types vary from hundreds to tens of thousands in MCA dataset, to avoid the under-representation of the small-sample-size cell types in the calculation of the accuracy function, we include the class-weight based on the sample size of each type in the model training. The loss function is defined as categorical cross-entropy.

### Organization and preprocessing of the training dataset

There are three versions of SuperCT discussed in this paper, v1m (‘m’ stands for mouse), v1h (‘h' stands for human cells) and v2m, which are adapt to the different application scenarios. We firstly select a total of 176 675 cells in the 30 categories of known cell types defined in mouse cell atlas project (MCA). These 30 cell types have more than 1000 counts in MCA. We then selected 4227 cells (4.2k-new-type-cells) in the seven categories of known cell types other than the 30 types in MCA. These seven cell types have >500 counts in MCA. We also synthesized 8923 scRNA-seq profiles (9k-synthetic-cells) by shuffling the randomly selected MCA scRNA-seq profiles. Total 189,825 labeled single-cell expression profiles (referred as the 190k training dataset) were used for the training of SuperCT v1m and v2m. To test the robustness of the model expansion from v1m to v2m, the variable training labels of the two versions are described as follow. Other than the 9k-synthetic-cells that are always assigned to ‘unknown’ category, the 4.2k-new-type-cells were assigned to the ‘unknown’ category in v1m but assigned to seven specific types in v2m. We are wondering whether the extra seven types could be learned from the ‘unknown’ category in the new training. Other than the abovementioned MCA and synthesized cells, 8976 additional peripheral blood mononuclear cells (PBMCs, 9k-immune-cells) from 10× Genomics public database are included for SuperCT v1h training. In order to make a fair comparison, the 4.2k-new-type-cells were still assigned to the unknown category in v1h because v1h is considered as the optimization of v1m.

### Initial training and continual learning of the models

For the SuperCT v1m, we use the batch learning of the entire 190k MCA data. We applied SGD (Stochastic Gradient Descent) by dividing training data into mini-batches of size 1024. 20% of training dataset was set aside for the training validation. Numbers of training epochs were manually determined based on the gap between the training and validation error to control over-fitting (early stopping).

In the two upgrades from v1m to v1h and v1m to v2m, we took the v1m parameters to initiate the online learning. We also took the mini-batches of 1024 from the new training data and update the model parameters. In order to alleviate the ‘Catastrophic Forgetting’, we designed a ‘review training’ mechanism to ‘refresh’ the memory of the previous learning in the v1m. Randomly selected 4000 cells from the previous training dataset are fed again for the model training after each epoch of learning by the new training dataset.

### 10-fold cross-validation over the training datasets and the model

To evaluate the overall performance based on the total training dataset (190K MCA and 9K human immune cells), we performed 10-fold validation test. The v1m, v1h and v2m datasets are randomly divided into 10 equal fractions. Nine of them are used to perform training and one of them is held out for testing. The 10 concordance values will show the robustness of the model and the reliability of the training datasets.

### Transfer learning in the model expansion

In the training of the new cell types using MCA dataset, we freeze the first hidden layer (200 nodes) and only update the weights of the second layers and the output layer. This learning strategy will retain the transformed features residing in the hidden layer in previous training and use them for the characterization of more cell types.

### Testing datasets from the third party

The first testing dataset is based on the similar study as MCA, which was performed by the Tabula Muris Consortium (TMC) (14). We used the single-cell expression profiles from 12 tissues generated on the 10xGenomics Chromium platform in order to test the overall performance of the models trained by MCA cells. The MCA and TMC cell types are defined by two different groups of investigators. To make a fair comparison, we first identified and selected the TMC cell types that are corresponding to the 37 known cell types defined in SuperCT (see the mapping in Supplementary Table S1). The TMC cell types whose MCA-corresponding type not confirmed are excluded for now. We have total 25.5k cells in this test.

The second testing dataset was from cord blood mononuclear cells (CBMC, total 8005 cells). Stoeckius *et al.* used a unique CITE-seq technique that allows to interrogate the single-cell transcriptome and surface proteins at the same time (15). The cell identities revealed by CITE-seq technique are validated by both RNA and surface protein signals.

The third testing dataset is obtained from an E18 mouse brain 9k cells generated on the 10× Genomics platform (16). This dataset will cover some new cell types that are defined only in the upgraded model. We use this dataset to test the robustness of the expansion of cell-type catalog.

The fourth testing dataset was a 2.8k-tumor-tissue-associated-cells dataset obtained from a malignant pancreatic tumor tissue that was extracted from a genetically modified mouse (the KPC model)(17) generated on the Chromium platform. The study of the third dataset represents a typical scenario in which researchers seek to extensively interrogate a heterogeneous tissue sample using the scRNA-seq technique.

### scRNA-seq dataset generated from mouse pancreatic tumor tissue

Freshly harvested tumors from KPC (LSL-KrasG12D/+; LSL-Trp53R172H/+; Pdx-1-Cre) mice were subjected to mechanical and enzymatic dissociation using a Miltenyi gentleMACS Tissue Dissociator to obtain single cells. The 10xGenomics Chromium Single Cell 3′ Solution was employed for capture, amplification and labeling of mRNA from single cells and for scRNA-seq library preparation. Sequencing of libraries was performed on an Illumina HiSeq 2500 system. Sequencing data (fastq files) was input into the CellRanger pipeline to align reads and generate gene-cell digital expression matrices.

### Unsupervised clustering, dimensional reduction, and data visualization

Most of the UC, DR and DV in this paper are accomplished by a widely used scRNA-seq analytical suite, Seurat (17,18). The Seurat objects are generated for each dataset with their digital expression matrices as input. The PCA is performed by Seurat RunPCA function. The tSNE coordinates are calculated using Seurat RunTSNE function. The putative clusters are defined by Seurat FindClusters function using the top 10 principle components and other default parameters. The prediction result is loaded to the Seurat metadata in order to be shown in reference to the tSNE layout.

### Top signature genes for each cell type ranked by information gain

In order to calculate and rank the information entropy gain, the following equations are used.

(1) \begin{eqnarray*}Gain\ (Type,\ gene) &=& \ Entropy(Type) \nonumber\\ &&- Entropy(Type,\ Gene)\end{eqnarray*}

(2) \begin{equation*}Entropy\ (type) = - \mathop \sum \limits_i {p_i} \cdot \log \left( {{p_i}} \right)\end{equation*}

(3) \begin{eqnarray*}Entropy\ (Type,Gene) = \ - \sum\limits_i {({p_i} \cdot \sum\limits_j {{p_{ij}} \cdot \log ({p_{ij}})} )}\nonumber\\ \end{eqnarray*}

The binary values of each gene over the binary values as a certain type for the total cells are utilized to make a contingency table in order to calculate the *p* in Equations (2) and (3). Whereas pi denotes the prior probability of cell belonging to the cell type *i*; *p*_*ij*_ denotes the probability of cell belonging to the cell type *i* (yes or no) when gene signal is in *j* status (present or absent). The top 50 genes are ranked based on the value of information gain (Equation 1) in Supplementary Table S2 for each type.

### Search of genes that correlate to the tumor progression

The epithelial tumor cells are characterized from the scRNA-seq data of the mouse PDAC tumor tissues using SuperCT v2m. These cells’ digital expression profiles were pulled out as the input to calculate the pseudo-time ordering using Monocle2. The pseudo-time values of each cell and the gene expression value for that cell were used to fit a linear model using an R function ‘lm’. The *p* value of the coefficient of each linear model is used to determine whether the gene expression correlates with the tumor progression by time.

## RESULT

### Overview of SuperCT model structure, datasets, training strategies and usage

**Figure 1.**
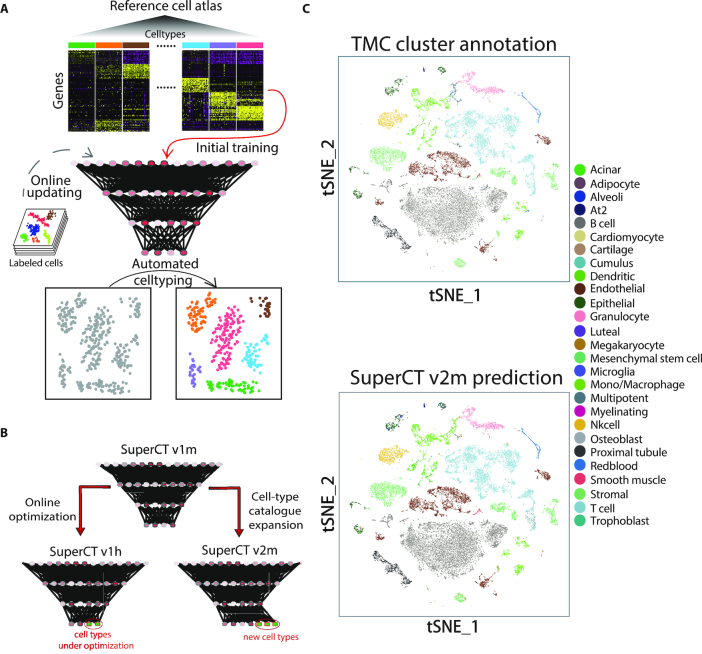
Overview of SuperCT framework and the high concordance between the original MCA cell-type annotations and the SuperCT predictions. (**A**) The workflow of SuperCT training, prediction and upgrade. (**B**) The two upgrades that lead to the optimized or expanded SuperCT classifier. (**C**) The overview of TMC data original labels in comparison with SuperCT v2m predictions.

The schematic workflow of SuperCT framework is shown in Figure 1A. The detailed structure of the fully connected ANNs for SuperCT is described in the Materials and Methods. The training datasets were selected from MCA project (∼190k cells) and peripheral blood mononuclear cells (PBMCs, ∼9k cells). More details of the training data organization are given in the Materials and Methods and Supplementary Table S3. Other than the training dataset, in order to further validate the utility of SuperCT classifier in assigning cell types, we tested the performances using three independent datasets (14,19). More detailed descriptions of these datasets are given in the Materials and Methods. The overall concordance measure is defined to assess the reliability of the prediction. More precisely, concordance means whether the SuperCT predictions agree with the manual cluster annotations in the literature, which were based on the enriched canonical marker of the entire cluster of a certain sample or the entire cellular sample sorted by the canonical markers from the specific tissue.

There are three versions of SuperCT classifier discussed in this paper, v1m, v2m and v1h. v1m and v1h both have 30 ‘known’ and one ‘unknown’ cell type in their catalogs. v2m has 37 ‘known’ and one ‘unknown’ type. ‘m’ stands for mouse and ‘h’ stands for human, which indicates on what species the model works better. Version number increase stands for the upgrade of the cell-type catalog. Figure 1B illustrates the two upgrade protocols that underlie the typical evolution of SuperCT model. v1m is considered as the initial model trained from a large training dataset possibly with some error or insufficiency. In the v1m-to-v1h upgrade, we aim to test whether the online parameter optimization using additional training dataset from the similar background will make the classifier perform better in the target sample. In the v1m-to-v2m upgrade, we aim to demonstrate the robust expansion of the capability to predict more cell types with little sacrifice on the concordance over the predictions of other cell types.

**Figure 2.**
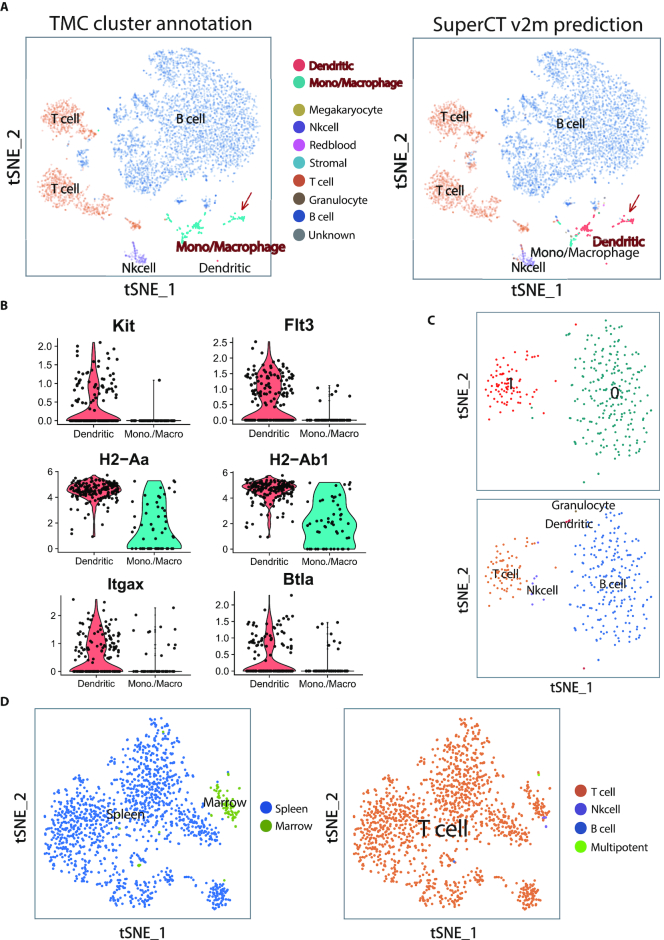
The outperformance of SuperCT over traditional UC-based methods. (**A**) Discordant cell labels by TMC and by SuperCT v2m predictions in spleen tissue: monocyte/macrophage versus dendritic cell. (**B**) The higher signal of dendritic cell signature genes suggests the SuperCT gives more convincing labels. (**C**) Down-sampling makes the minor cell populations lose the power to form discernable cluster but SuperCT can still characterize the cell type. (**D**) The separated clusters of the same cell types derived from batch difference can still be correctly characterized by SuperCT.

We used another mouse cell study (TMC datasets) to validate the overall performance of v2m model (Figure 1C). The outperformance of SuperCT classifier over the prior manual annotation based on UC methods will be compared and discussed in detail in the following result section (Figure 2).

Using a 2.3 GHz CPU processor computer, the runtime of SuperCT training on 190k cells is ∼20 s per epoch. It usually takes less than 10 epochs to achieve convergent and concordant result, which means overall training time is less than 5 minutes. Using the same computational resources, SuperCT is able to characterize ∼10 000 single-cell expression profiles in <1 min, whose prediction efficiency is far superior to traditional UC-methods that requires a lot of interactive operation and domain knowledge as input.

### The initial, optimized and expanded models of SuperCT

**Figure 3.**
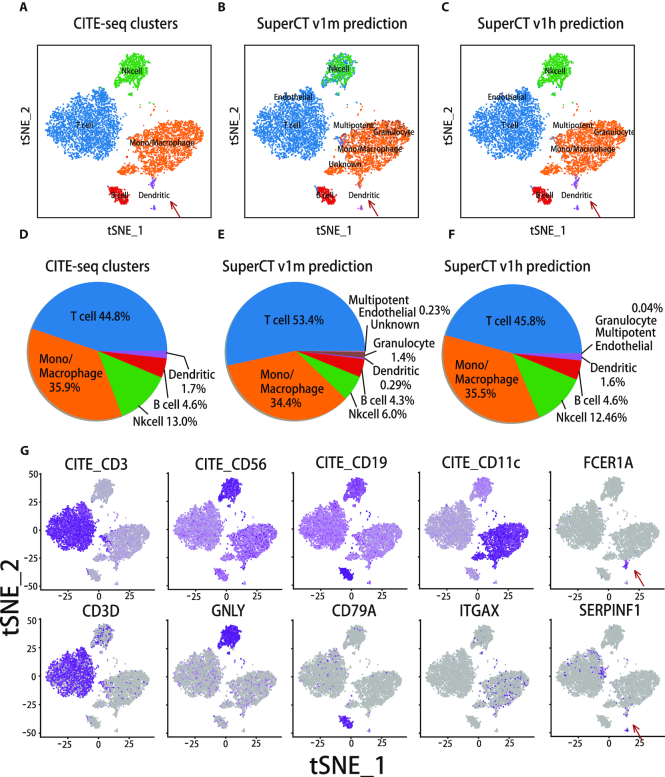
The upgraded predictions of CITE-seq human PBMC cells that are confirmed by the marker signals of both surface proteins and RNAs. (**A**) The original cluster labels on the tSNE layout of CITE-seq PBMC cells. (**B**) The predictions by SuperCT v1m. (**C**) The predictions by SuperCT v1h. (**D**) The fractions by the original CITE-seq PBMC cell types. (**E**) The fractions of the SuperCT v1m cell-type predictions. (**F**) The fractions of SuperCT v1h cell-type predictions. (**G**) The signal distributions of the canonical marker genes for the PBMC subpopulations that are concordant to the SuperCT predictions.

We carefully examined the v1m validation results in reference to the original MCA cluster annotations using the confusion matrix across the defined cell types. The true cell-type calls go to the diagonal of the matrix. Other than the undefined seven cell types that go to ‘Unknown’ category, it is found that a few known cell types show lower concordance than the others (see confusion matrix in Supplementary Figure S1A). Low concordance suggests the unsatisfactory performance of discerning these specific known types in the v1m training, most of which were difficult to annotate at the first place because of many shared markers. Even though the erroneous labels in MCA will compromise the training effect, it was still difficult to correct the original annotation without additional information to reach the ground truth in such large scale of data as MCA. Besides, in a testing dataset from human PBMC (CITE-seq), we found that v1m prediction result is at low concordance (88.3%), which is yet to improve (Figure 3A). Other than the possible erroneous labels in the training dataset, the subtle discrepancy of the molecular signatures of immune cell types between mouse and human may also explain the low performance. Altogether, we believe the online optimization using higher confidence training data is necessary and can tackle the problem. Given the availability of the datasets for a few of these types from another public resource (16), we were able to input more human immune cells from the tissue with higher confidence on the identity of human immune cells (peripheral blood) and thus to enhance the feature learning. This one is called v1m-to-v1h upgrade.

In the model upgrade, instead of redoing the entire MCA 190k dataset in combination with the new PBMC 9k dataset, we implemented a more efficient ‘online learning’ strategy in the v1m-to-v1h upgrade. In online learning, ‘Catastrophic Forgetting’ is a tendency of ANN that the new training could make the model lose the ‘memory’ of the old training. This issue has been also taken care of using a ‘review training’ mechanism. The details of both strategies are described in the Materials and Methods. We used the CITE-seq PBMC dataset whose background is similar to the feature enhancement training dataset to assess the outperformance of v1h over v1m whose result is shown in Figure 3 and explained later.

In the v1m-to-v2m upgrade, we specifically incorporated the ‘transfer learning’ strategy, which means some of the model parameters can be re-used in the new categorization tasks of the expanded reference catalog. It is believed such a strategy can improve the training efficiency by transfer knowledge from previous learning tasks (20). More details of implementation are given in the Materials and Methods. The high concordance (91.4%) between v2m predictions to the original TMC annotation shown in the following results suggests the success of transfer learning in cell-type characterization task.

**Figure 4.**
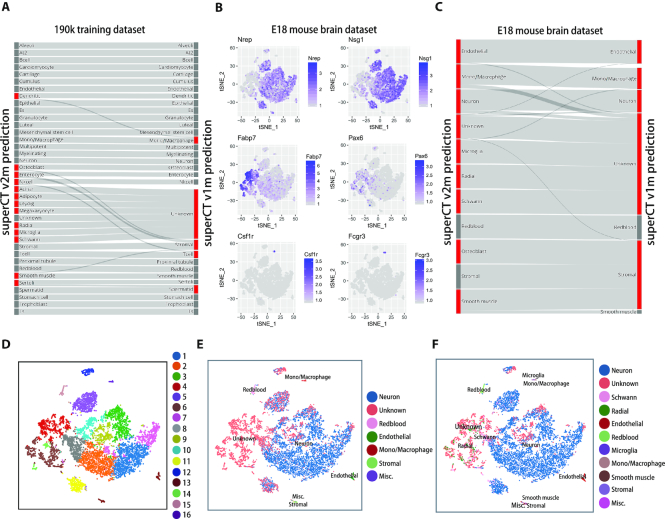
The assessment of the v1m-to-v2m upgrade by comparing the SuperCT predictions of different version in both 190k training dataset and E18 mouse brain dataset. (**A**) The Sankey diagram showing the upgrade of SuperCT v2m (left) labels from SuperCT v1m (right) in 190k training dataset. (**B**) The signal of the signature genes indicating the corresponding cell types of different clusters in E18 brain dataset. (**C**) The Sankey diagram showing the upgrade of SuperCT v2m (left) labels from SuperCT v1m (right) in the 9k E18 brain dataset. Most of the new cell types are from ‘unknown’ types. Some of the Microglia are from mono/macrophage type and some are from ‘unknown’ category. (**D**) Unsupervised clusters of 9k E18 brain dataset. (**E**) SuperCT v1m predictions of 9k E18 brain dataset. (**F**) SuperCT v2m predictions of 9k E18 brain dataset.

The v1m-to-v2m upgrade is nontrivial because it demonstrates how to make the SuperCT characterize more cell types when more cell types of training datasets are available. For the 190k MCA dataset, SuperCT v1m achieves 97.3% (171 677 cells) of concordance to the original labels in the 30 ‘known’ type category (176 675 cells). SuperCT v2m achieves 96.6% (174 739 cells) of concordance in the 37 ‘known’ type category (180 888 cells). Although the concordance rate seems a little bit lower in the v2m, total 3,062 more cells’ identities were accurately revealed (from unknown to specific known type) in the same dataset by the v2m. Both v1m and v2m give 100% concordant result to the MCA cells in the ‘Unknown’ category. In the meantime, Sankey diagram (Figure 4A and C) shows this upgrade has limited influence on the characterization of other known types of v1m in the third party, which suggest the learning of the new cell types does not make the model ‘confused’ over the other ones. In conclusion, the v2m does accurately characterize more cell types, thus outperforms v1m.

To ensure the robustness of the three versions of SuperCT algorithm and the reliability of the training datasets, we performed 10-fold cross-validations on the training datasets and the average ∼92% of concordances on the 10% hold-out testing datasets show the satisfactory outcomes (see detailed descriptions in Materials and Methods and in Supplementary Table S4).

To ensure the SuperCT accuracy that is not a result of over-fitting, we use 25,515 TMC cells independent of the training datasets for prediction. The corresponding cell-types in SuperCT v2m for TMC labels are linked and cross-compared in this dataset. Figure 1C shows the predictions of SuperCT v2m colored by the cell types, which share the same color-code as original TMC cluster annotation. The overall similar color scheme shows the high concordance. The concordance among the 37 known cell types is as high as 91.4%, which means SuperCT gives the overall accurate predictions. Moreover, the careful examination on the markers of some of these clusters indicates the SuperCT accuracy is even higher because TMC mislabeled some of the cells (result shown in the next section). Supplementary Figure S1D shows the concordance of predictions for each to the 37 types.

### SuperCT solves the limitations of unsupervised clustering methods

We demonstrate that SuperCT overcomes the three challenges that are commonly seen when unsupervised clustering is performed.

Challenge A: the unexpected cell type can be easily mislabeled to the similar one due to the lack of familiarity of the specific markers. Figure 2A shows some of the TMC macrophage cells in a mouse spleen are labeled as dendritic cells by SuperCT. Figure 2B shows the signal distribution of the genes over two cell types predicted by SuperCT, which suggest SuperCT most likely gives the correct answer on them. These dendritic-cell markers were probably not examined by the TMC investigators.

Challenge B: Small number of cells of a certain type won’t be easily characterized by UC. Figure 2C shows two putative clusters defined by the default Seurat unsupervised clustering parameters after down-sampling of the same spleen tissue dataset (randomly selected 300 from 6113 cells). We compared the original TMC labels, SuperCT predictions, and the UC result. The tSNE layout shows the distribution of the minor cell type identified in the original dataset (*n* = 6113, shown as Figure 2A) cannot form an obvious cluster in the down-sampled dataset (*n* = 300, shown as Figure 2C) that makes the minor cell population very hard to discern. Nonetheless, the SuperCT can still accurately characterize the small-population cell types that are concordant to the TMC labels defined before downsizing, no matter how small the cell count is or how the tSNE layout looks like.

Challenge C: Figure 2D shows the tSNE layout of the T cells (originally defined by TMC) extracted from two immune tissues, spleen (sample id: P7_6, *n* = 1353) and bone marrow (sample id: P7_2, *n* = 90). The biological and/or technical variability leads to observable cluster separations in two tissues (Figure 2D, left panel). The SuperCT gives 1435 T cells predictions (99.4% concordance) with lease impact of the cluster separation (Figure 2D, right panel). Nonetheless, the technical or the biological difference that drives the cluster separation needs to be figured out.

In summary, the SuperCT prediction is independent of the result of clustering or human interpretation. Neither insufficiency of cells nor batch effect impedes the accurate characterization of the cell types.

### Cell-type-specific signature genes defined by SuperCT

As we know, each cell type has multiple signature genes that dictate its function and reveal its identity. Although SuperCT did the classification work in a ‘blackbox’, we were still able to uncover the signature genes by ranking the information gain of each gene's presence/absence in discerning a cell type from the other types (More details are given in the Materials and Methods). The top 50 genes for each of 37 cell types are shown as examples in the Supplementary Table S2. These top-rank genes provide the clue to explore the unique molecular features that underlie the v2m 37-cell-type categorization. Some of the top-rank genes, such as CD3D for T cells, CD79A for B cells, KDR for endothelial etc., are very familiar to us, though many others have never been reported previously which provides the new insights into these cell types.

### SuperCT predictions on CITE-seq dataset

For the CITE-seq CBMC dataset, Figure 3A–C shows the layout of four predominant clusters, one minor population hidden at the lower corner (classical dendritic cells) and one minor segregated cluster (plasmacytoid dendritic cells). The original paper defined these five cell clusters based on their surface marker signals and the signature RNA signals. Figure 3B shows the characterizations by SuperCT v1m. The overall concordance is 88.3%. Figure 3C shows the characterizations by SuperCT v1h. The overall concordance is 98.5%. Supplementary Figure S1e gives the details of the concordance of each predicted cell type on v1h. Figure 3D–F shows the percentage of the annotated cell types. Figure 3G shows more details of the marker signal of both surface proteins (top row) and RNA transcripts (bottom row) for the five cell types. The result suggests v1h gives the predictions with higher concordance than v1m in CITE-seq PBMC dataset.

### SuperCT predictions on E18 mouse brain dataset

In order to specifically validate the expandability of this SuperCT framework, we carefully examined the change of the prediction result before and after the model upgrade in MCA training dataset and E18 mouse brain dataset. For the 190k training dataset, the Sankey diagram, Figure 4A shows that the seven new cell types defined by v2m were mainly derived from the ‘unknown’ types as defined by v1m. In E18 mouse brain dataset, three out of seven additional cell types, (Radial glia, Microglia, and Schwann cells) in v2m which are nervous-system-related were anticipated in the mouse brain tissue. The Sankey diagram for this dataset, shown in Figure 4C, provides the details on how the SuperCT v2m have learned the three new cell types from the ‘unknown’ classes or the similar cell types (microglia versus mono./macrophage) from v1m. This result suggests, by increasing the output nodes of ANN model and including sufficient new training cells, SuperCT is able to predict more specific cell types. Interestingly, v1m made an ‘excusable’ mistake by taking microglia cells as monocyte/macrophages (Figure 4A, C and E). This ‘mistake’ can be explained by the similar roles of microglia to the nervous system as the macrophage to the other part of a mammalian body. Figure 4B and Supplementary Table S2 also shows multiple genes, such as colony-stimulating factor receptor (CSF1R) and Fc fragment of IgG receptor (FCGR3A), are shared by mono./macrophage and microglia cells. Figure 4E and F shows the tSNE layout of the predicted cell types by v1m to v2m, suggesting the successful evolution of the model predictions from unknown to specific cell types that gradually uncover the role of the unsupervised cell clusters in the Figure 4D.

### SuperCT predictions on the KPC mouse tumor dataset

**Figure 5.**
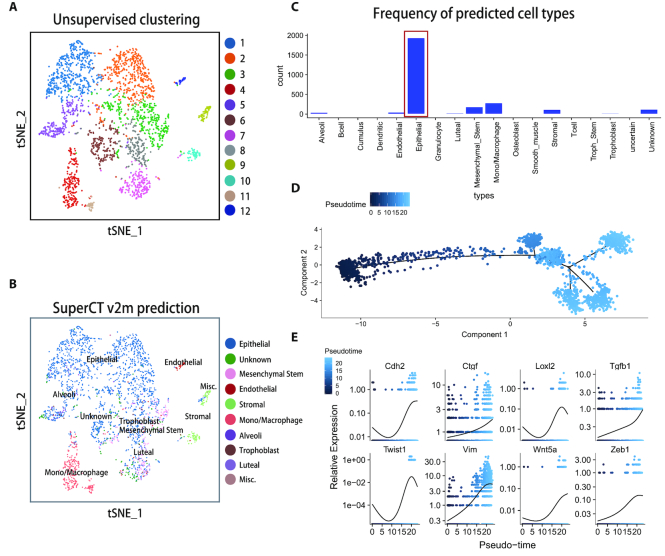
De-convolution of the signal of the constituent populations of mouse pancreatic tumor tissue. (**A**) The unsupervised cell clusters of the mPDAC tissue defined by Seurat FindClusters function. (**B**) The predicted cell types of the mPDAC tissue. The minor types with less than five cells are not shown in color legend. (**C**) The fractions for the cells of the predicted cell types in mPDAC. The epithelial tumor cells in the red box are sorted out for the downstream analyses. (**D**) the pseudo-temporal trajectory of the tumor progression based on sorted tumor cells plotted by Monocle 2. (**E**) The EMT genes display up-regulations and correlate to the tumor progression.

At last, we summarize the SuperCT result of a pancreatic ductal adenocarcinoma dataset derived from KPC mouse (mPDAC, dataset #3). Figure 5A shows Seurat FindClusters function yielded 12 putative cell clusters while SuperCT characterized total 17 cell types (Figure 5C). We were particularly interested in the primary epithelial tumor cells, which had been assigned to ‘Epithelial’ type by SuperCT v2m (Figures 4C and 5B). We then perform the *in silico* ‘sorting’ of the predicted 1940 epithelial tumor cells. The typical mPDAC markers, e.g. EPCAM, cytokeratin 19, CD133 etc., were indeed enriched in these cells, validating the tumor cell identity (Supplementary Figure S3). With the cell identity information brought by SuperCT v2m, we were able to further explore whether any dynamic change of the molecular signals could be observed in the predicted epithelial tumor lineage. The pseudo-temporal analysis was performed on these ‘sorted’ tumor cells using Monocle 2 method (21). A tumor progression trajectory was thus derived. The order information of the tumor cells in the inferred trajectory was utilized to perform the regression analysis in order to reveal the relationships between the tumor progression and the gene expression change. Supplementary Table S5 shows a list of genes that give significant *P*-values for the regression coefficient. (See details in the Materials and Methods). A number of the correlated genes, such as N-cadherin, Twist1, Loxl2 etc., that are involved to the epithelial-mesenchymal-transition (EMT) were identified. (shown in Figure 5E) Other than the epithelial tumor population we have extensively discussed as above, the clusters C4+C11, C9+C10 and C12 defined by Seurat FinderClusters function are corresponding to the SuperCT predictions of monocyte/macrophage, stromal and endothelial cells (Figure 5A and B). In here, SuperCT gives explicit interpretation on these clusters.

### Miscellaneous

In comparison between UC cluster and SC predictions, it is found many of the unsupervised clusters determined by multiple top principal components (distance metric implemented in Seurat) can be explained by SuperCT predictions, which suggests the subpopulations segregated in the high dimensional space could correlate to the previously defined cell types. Even though some of the clusters might need to be further explored (Supplementary Figures S2, S3D–F, S4A, and B).

It is also notable, the MCA dataset was generated on the Microwell-seq platform and the 4.2k PBMC dataset was generated on the Chromium scRNA-seq platform (10× Genomics). Three testing datasets are generated on the platforms of CITE-seq, and Chromium scRNA-seq platforms, respectively. All these platforms produce the typical digital expression matrices based on UMI counts. Moreover, CITE-seq PBMC dataset was generated from human tissue whereas the MCA, E18 brain and mPDAC datasets were generated from mouse tissues. The reliable results suggest the compatibility of SuperCT framework is not only across different scRNA-seq platform but also across different mammalian species, though the online optimization like v1m-to-v1h upgrade would benefit the application of the specific scenario.

## DISCUSSION

In this study, using training and independent testing datasets, we have demonstrated the superior performance of the supervised-learning-based classifiers in the characterization of the previously defined cell types in the heterogeneous tissue samples. This is the first scRNA-seq analytical framework that is independent of unsuperversied clustering. With least requirement of the expertise or the bioinformatics skillset from the individual user, the SuperCT classifier delivers accurate cell types information for thousands of single-cells in just a few minutes.

A classifier with good performance is not built overnight. Neither is it a one-size-fit-all model. It is actually the growing human knowledge that drives the machine learning, which means a continual learning mechanism of this framework should be designed at the very beginning. This is also a big advantage of SuperCT framework over the other existing solutions. The two successful upgrades described in this paper have proved that the online-learning and the transfer-learning algorithm make the model evolution not only possible but also efficient.

Before the human cell atlas data is generated and released, we don’t have the comprehensive training dataset resources to cover too many human cell types. In this study, we have found that the SC model trained from the mouse cell types is also applicable to human cells in most of the predictions. This is not a surprising result because human and mouse do share many homologous genes and they contribute to the similar functional cells with the overall similar molecular signatures. It is believed that a general model trained from a typical mammalian species should be applicable to another mammalian species. This finding will expand the scope of the application when the training data is in short for a certain species. Nonetheless, it is intuitively believed that the cell-type classifier should work better when the training data is from the same background.

Supervised and unsupervised methods play the complementary roles in the growing findings facilitated by machine learning. The classification results from both methods in the analyses of scRNA-seq data can be the cross-reference to each other. As it has been observed, many of the cell grouping defined by UC can be validated by SC. The cell grouping by SC takes the full advantage of the *a priori* learning that makes the cell type prediction less subjective but more efficient. The UC cell grouping is driven by the current data, whose new information could lead to the new findings. Three possible scenarios in UC and SC comparison should be further explored in order to achieve new findings. (i) The cells form segregated clusters by a UC method with cluster-specific molecular features but SC characterization may give only one cell-type. (ii) There is an obvious cluster, which may be categorized as ‘unknown’ by SC. (iii) There is a cell type that is characterized in the tissue that may ‘not make any sense’, such as the ‘monocytes’ were found in mouse brain by SuperCT v1m.

## DATA AVAILABILITY

The web app for different versions of SuperCT can be accessible from the following URL. https://sct.lifegen.com/. The accession number for the KPC mouse PDAC tumor data shown in this paper is NCBI GEO: GSE126388. Sample Y00029 is what has been used in this paper.

## Supplementary Material

Supplementary Data
