# Supplementary material for: SuperCT: a supervised-learning framework for enhanced characterization of single-cell transcriptomic profiles
Source: Nucleic Acids Res. 2019 Feb 25;47(8):e48. doi: 10.1093/nar/gkz116 (PMC6486558; doi:10.1093/nar/gkz116)
Supplement: Supplementary Data [file gkz116_supplemental_files.zip › Supplementaries_final_after_acceptance.docx]

Supplementary Table 1:

The mapping between TMC annotation and SuperCT cell types.

Supplementary Table 2:

The top 50 signature genes with the most information gain in discerning each cell type by SuperCT v2m.

Supplementary Table 3:

The cell types and the MCA dataset for 190k cells used in the SuperCT model training.

Supplementary Table 4:

The concordance indices of the 10 fold cross-validation for the three versions of model and their training datasets.

Supplementary Table 5:

The genes that significantly correlate to the tumor progression.

Supplementary Fig. 1

The confusion matrices of SuperCT predictions over original MCA, TMC and CITE-seq CBMC cell-type annotation.

Supplementary Fig. 2

Unsupervised clusters and the v1h predictions on CITE-seq dataset.


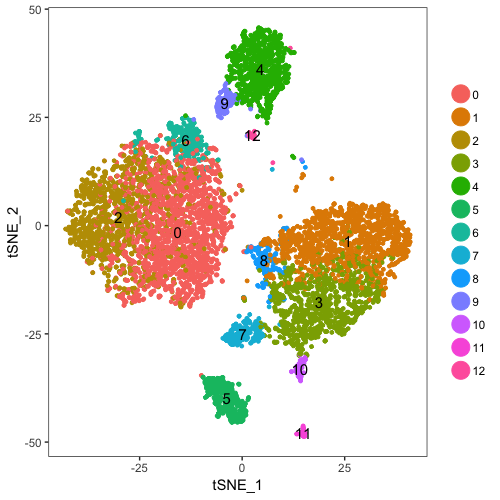

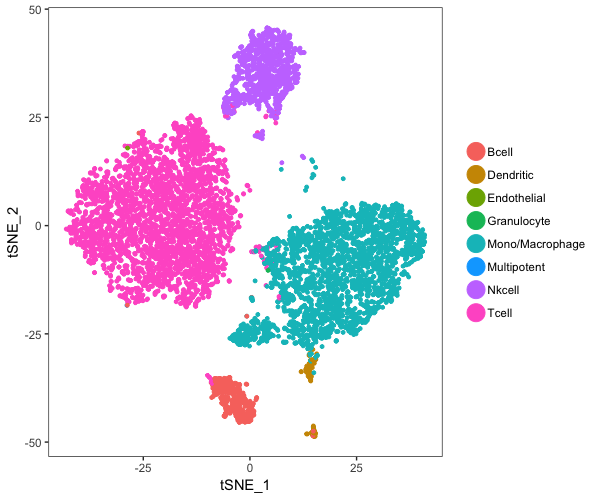


Supplementary Fig. 3

The signal distribution of the signature genes for the predominant cell types in mouse PDAC sample.
